# Supplementary figures and images for: Biparental contributions of the H2A.B histone variant control embryonic development in mice
Source: PLoS Biol. 2020 Dec 23;18(12):e3001001. doi: 10.1371/journal.pbio.3001001 (PMC7757805; doi:10.1371/journal.pbio.3001001)

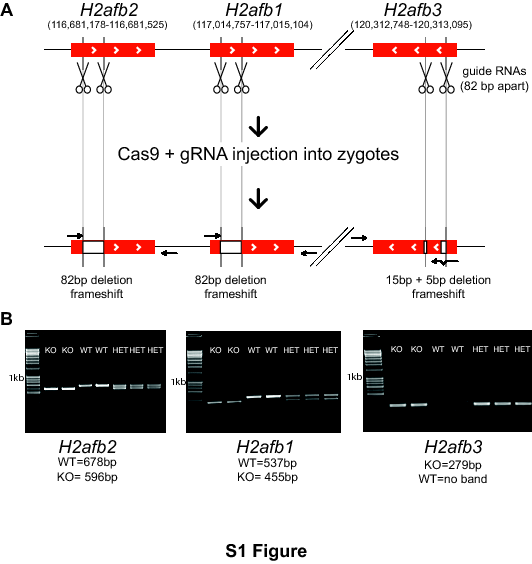

Supplement: S1 Fig — (A) Schematic of the CRISPR/Cas9 strategy used to produce ΔH2A.B animals. All H2afb coding loci mapped in mm10 are shown with their coordinates in parentheses. Details on the sequenced deletions for each paralog are shown at the bottom. Scissors indicate the regions of CRISPR/Cas9-induced cuts. Arrows indicates the location of genotyping primers (see Materials and methods for details) (B) A PCR genotyping example of ΔH2A.B alleles from pups of a HET × WT mating. A 1-kb plus ladder is shown on the left side of each gel. For H2afb1 and H2afb2, primers amplify a (smaller) KO or (larger) WT fragment. H2afb3 primers are specific for the KO allele and do not amplify a band in the WT. HET, heterozygous; KO, knockout; WT, wild-type. (TIFF) [file pbio.3001001.s001.tiff]

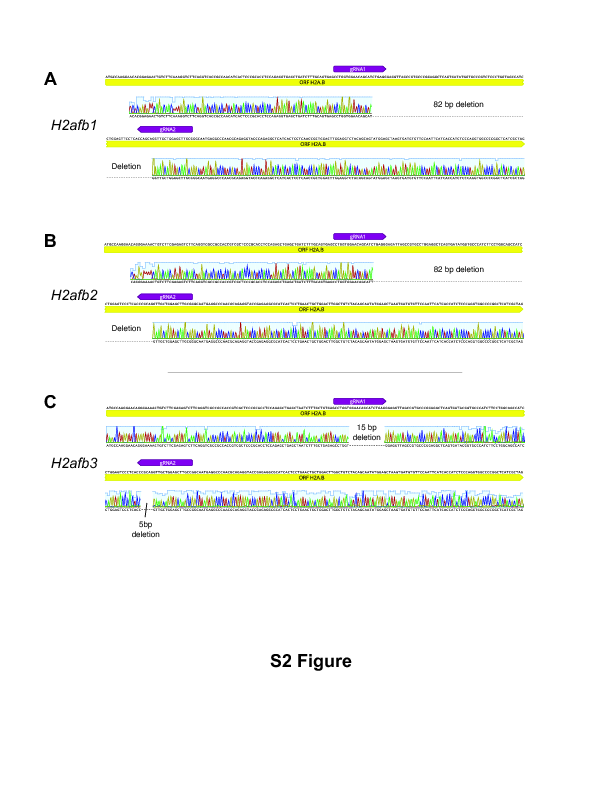

Supplement: S2 Fig — Alignment of H2afb paralog ORFs with Sanger traces from the sequencing of H2afb1 (A), H2afb2 (B), and H3afb3 (C) from ΔH2A.B animals. Annotated are ORFs (yellow boxes) gRNAs (purple arrow) and deletions. gRNA, guide RNA; KO, knockout; ORF, open reading frame. (TIFF) [file pbio.3001001.s002.tiff]

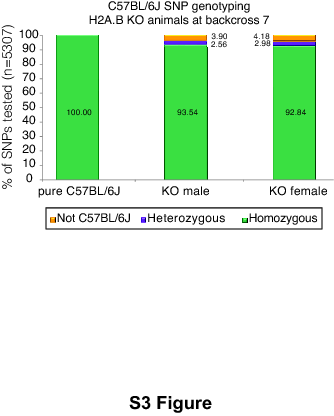

Supplement: S3 Fig — Percentages of HET, homozygous, or non-C57BL/6J SNPs in pure WT, H2A.B triple KO males or females are shown (Materials and methods). HET, heterozygous; KO, knockout; WT, wild-type. (TIFF) [file pbio.3001001.s003.tiff]

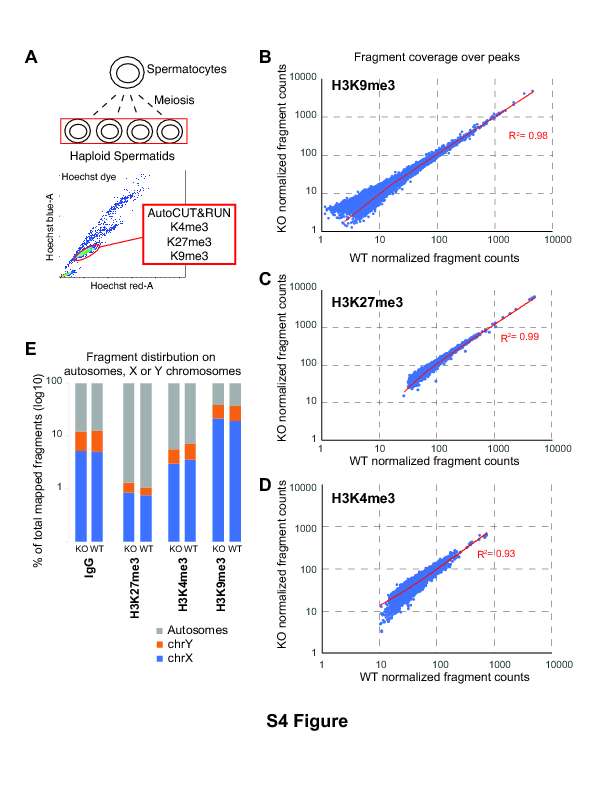

Supplement: S4 Fig — (A) Schematic of the flow cytometry (scatter plot) and MNase-based profiling (red box) used in this study. (B, C, D) AutoCUT&RUN normalized fragment counts (blue dots) over all peaks called for the histone modifications H3K9me3 (A), H3K27me3 (B), and H3K4me3 (C). KO counts are shown on the y-axis and WT on the x-axis. Linear regression and R2 values are shown in red. (E) Percentage of total mapped fragment assigned to chromosome X (blue), Y (orange), or autosomes (blue) (see S5 Data). Y-axis is in log10 scale. Results for fragments released by AutoCUT&RUN with IgG, H3K27me3, H3K4me3, and H3K9me3 are shown for both KO and WT samples. KO, knockout; MNase, micrococcal nuclease; WT, wild-type. (TIFF) [file pbio.3001001.s004.tiff]

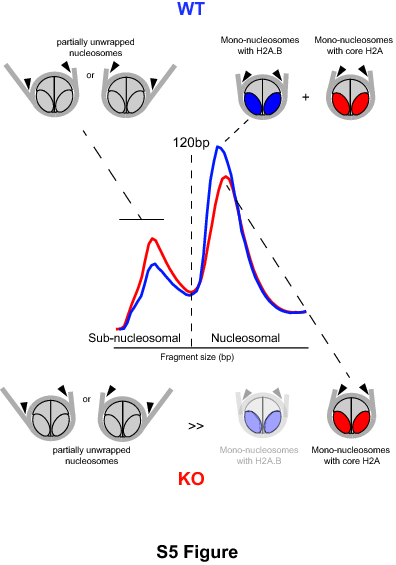

Supplement: S5 Fig — Schematic of fragment size (x-axis) distributions (y-axis) in WT and KO spermatids (replotted from Fig 1A). Sub-nucleosomal fragments below 120 bp are thought to correspond to partially unwrapped nucleosomes. In WT (blue), most nucleosomal fragments (above 120 bp) have the size of H2A.B containing mono-nucleosomes. In H2A.B KO (red), nucleosomal fragments have the size of H2A containing mono-nucleosomes. Note that H2A.B nucleosomes have a smaller MNase footprints than H2A nucleosomes. MNase cleavage is indicated by arrowheads. KO, knockout; MNase, micrococcal nuclease; WT, wild-type. (TIFF) [file pbio.3001001.s005.tiff]

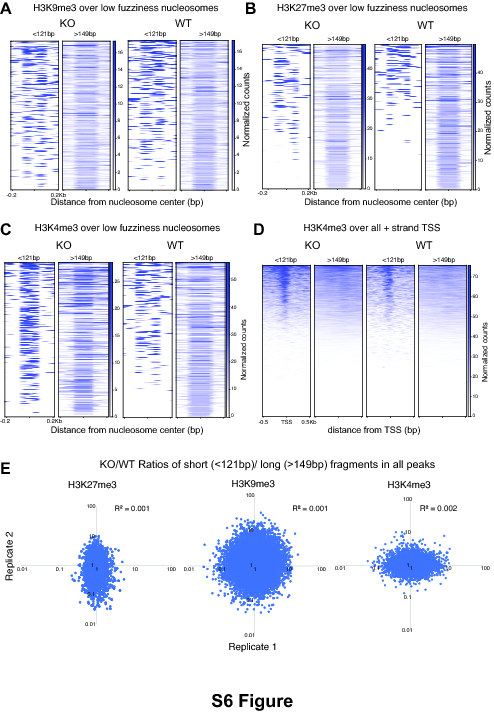

Supplement: S6 Fig — (A, B, C) Heatmaps of normalized counts (blue scale) of short fragments (<121 bp) and large fragments (>149 bp) over the top 2,000 low fuzziness nucleosomes in WT or KO samples are shown. Regions are centered on nucleosomes profiled with H3K9me3 (A), H3K27me3 (B), and H3K4me3 (C). (D) Distribution of fragments over the TSS of all + strand RefSeq genes. (E) KO/WT ratios of small/large fragment ratios in peaks defined by H3K27me3, H3K9me3, and H3K4me3 (see S6 Data). Log10 (ratios) for replicate 1 and replicate 2 are shown on the x- and y-axes, respectively. R2 values are shown in all 3 cases. KO, knockout; TSS, transcriptional start site; WT, wild-type. (TIFF) [file pbio.3001001.s006.tiff]

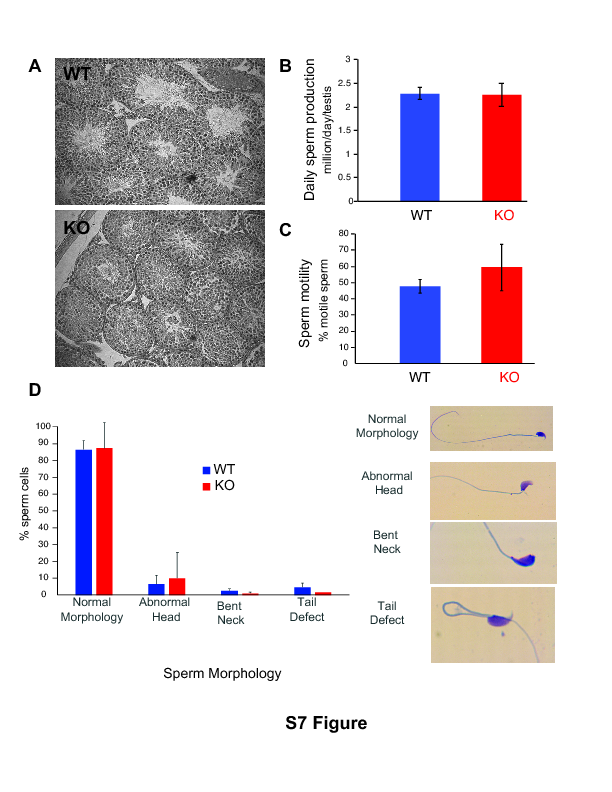

Supplement: S7 Fig — (A) Representative images of testis cross sections of WT and KO males (see Materials and methods) (B) Mean (with standard deviation, gray bars) DSP and (C) percentages of motile sperm measured from the same WT (blue) and KO (red) males are shown. (D) Fractions of sperm with normal morphology, abnormal heads, bent necks, or tails defects measured in WT or H2A.B KO males are indicated. Means and standard deviations are shown across replicates (WT n = 4; KO n = 3). Example images of each morphology are shown on the right. Supporting information can be found as S7 Data. DSP, daily sperm production; KO, knockout; WT, wild-type. (TIFF) [file pbio.3001001.s007.tiff]

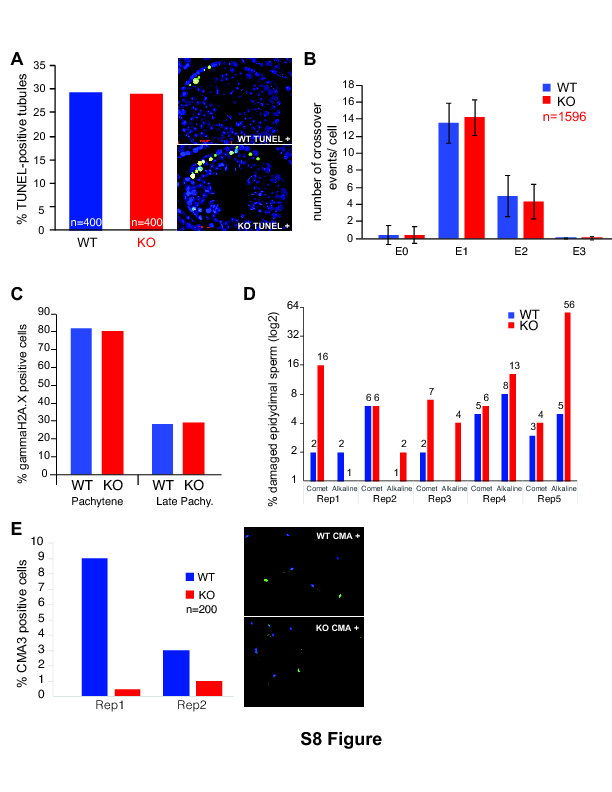

Supplement: S8 Fig — (A) Fraction of seminiferous tubules with at least 1 TUNEL positive cell in WT (blue) or KO (red) testis cross sections. n is the number of tubules counted. (B) Crossover events measured using staining against the proteins SCP3 and MLH1 (see Materials and methods) in germ cell spreads from WT (blue) and KO (red) animals. Mean (with standard deviation) number of chromosomes per pachytene cell with 0 (E0), 1 (E1), 2 (E2), or 3 (E3) cross overs are indicated. “n” is the total number of chromosomes counted for each genotype. (C) Fraction of gammaH2A.X positive cells at pachytene or late-pachytene in WT (blue) and KO (red) germ cell spreads. (D) Proportion (%) of epididymal sperm cells showing damage by Comet or alkaline diffusion (Alkaline) assays in WT (blue) or KO (red) samples. Individual replicates of WT and KO sibling pairs are shown separately (Rep1, 2, 3, 4, 5). (E) Fraction of CMA3 positive sperm cells in WT (blue) or KO (red) samples. Two replicates are shown. Two-hundred sperm were counted for each. Representative images of CMA3 positive cells are shown on the right. Supporting information can be found as S8 Data. CMA3, chromomycin A3; KO, knockout; MLH1, MutL homolog 1; SCP3, synaptonemal complex protein 3; TUNEL, terminal deoxynucleotidyl transferase dUTP nick end labeling; WT, wild-type. (TIFF) [file pbio.3001001.s008.tiff]

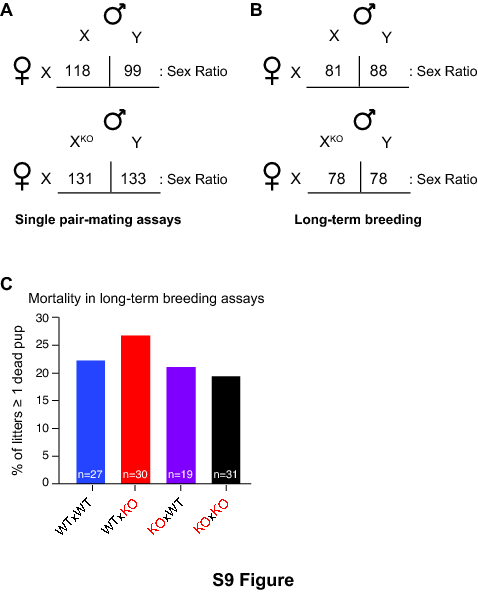

Supplement: S9 Fig — (A, B) Punnett squares of matings between WT females and WT males (top) or KO males (bottom) in fertility assays (A) or long-term breeding (B). The ratio of female pups (right squares) over male pups (lefts squares) denote the sex ratios. XKO denotes H2A.B KO chrX. (C) Percentage of litters with at least 1 dead pup in long-term breeding (see S9 Data). Mating is indicated as dam × sire. The total number of litters is shown inside each bar. chrX, X chromosome; KO, knockout; WT, wild-type. (TIFF) [file pbio.3001001.s009.tiff]

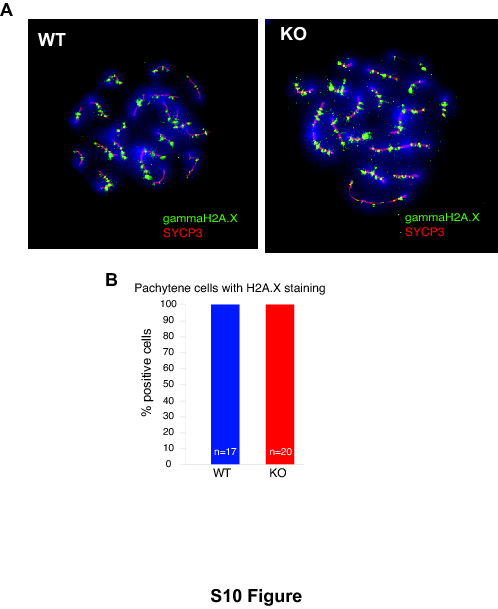

Supplement: S10 Fig — (A) Representative images of WT (left panel) and KO (right panel) pachytene cells, from E17.5 germ cell spreads (see Materials and methods), stained for gammaH2A.X (green) and SCP3 (red). (B) Percentage of gammaH2A.X positive pachytene cells in WT (blue) and KO (red) females (see S8 Data) and total number of cells counted are shown inside each bar. KO, knockout; SCP3, synaptonemal complex protein 3; WT, wild-type. (TIFF) [file pbio.3001001.s010.tiff]

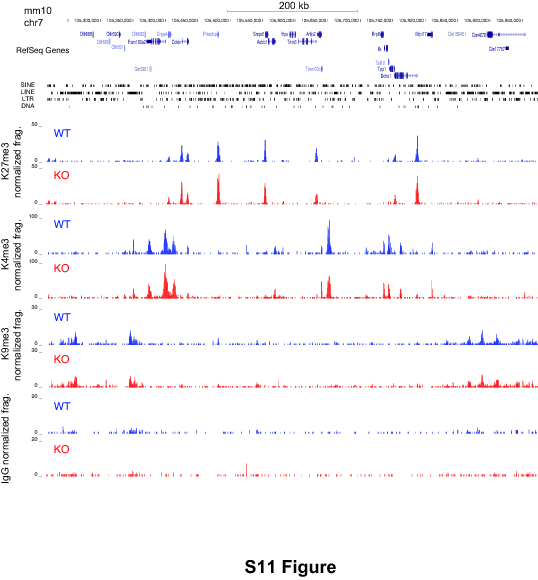

Supplement: S11 Fig — A segment of chr7 is shown, with Refseq genes, and transposon insertions (black bars). Normalized fragment counts across 2 replicates is shown for each AutoCUT&RUN dataset. WT samples (blue tracks) and KO samples (red tracks) are plotted for H3K27me3, H3K4me3, H3K9me3, and IgG. KO, knockout; WT, wild-type. (TIFF) [file pbio.3001001.s011.tiff]
