# Supplementary material for: Biparental contributions of the H2A.B histone variant control embryonic development in mice
Source: PLoS Biol. 2020 Dec 23;18(12):e3001001. doi: 10.1371/journal.pbio.3001001 (PMC7757805; doi:10.1371/journal.pbio.3001001)
Supplement: Attachment — Submitted filename: 106936_1_rebuttal_1863699_qgrtb7.docx [file pbio.3001001.s025.docx]

The authors would like start by thanking all three reviewers for their time, effort and extremely helpful comments. We have now added several experiments and have added additional details for previously described experiments to address the reviewers’ queries.

In particular, based on the reviewer suggestions, we have now performed and added four additional experiments to our analysis. Although it has taken us several months to perform these experiments (especially under Covid-related constraints) we believe these have substantially strengthened our paper:

1. We performed *in vitro* fertilization experiments that show that sperm from H2A.B KO males are fully competent for fertilization and resulting embryos do not have a higher degree of impairment compared to embryos sired from WT males. This experiment substantially adds to our analysis by narrowing down defects associated with H2A.B loss specifically to the post-implantation stage, in keeping with the ‘resource allocation’ hypothesis.
2. We performed chromomycin A3 (CMA3) staining as suggested by the reviewers; these analyses show that sperm from H2A.B males do not show gross deprotamination defects. Rather, we find that these sperm are hypercompact.
3. We performed more detailed characterization of spermatogenesis (‘clogged tubules’) and sperm defects and report these are not more substantial in H2A.B KO males compared to WT siblings. We also added more replicates for the DNA damage analysis of mature sperm and found a similar variability as initially reported.
4. We performed a preliminary characterization of female germ cell spreads and find no gross impairments upon loss of H2A.B.

We have also significantly revised our manuscript in three substantial ways based on the reviewer comments to improve clarity:

1. We now more extensively detail the genotyping strategy as well as details about the deletions present in our triple KO males. As you will see, even though we have not affected the transcriptional status of these H2A.B genes, none of them produce a protein that is more than third intact.
2. We now discuss in more detail the differences in phenotypes observed between our study and the previously published Anuar et al. study both in the results and discussion.
3. We now clarify the evolutionary model in response to the reviewer comments to make clear that H2A.B only partially meets the ‘demand-inducer/reducer’ model akin to genomic imprinting but otherwise satisfies the criteria for parental antagonism.

Point-by-point responses to each comment by the reviewer follow on the next pages.

**Point-by-point responses to Reviewers**

**Reviewer #1 (Evidence, reproducibility and clarity (Required)):**

****Summary**

In their paper, Molaro et al studied the consequences of H2A.B KO in mice. H2A.B is a histone variant chiefly expressed in male germ cells. It has been shown to accumulate in meiotic and post-meiotic male germ cells. H2A.B is encoded by 3 genes which have been shown to be under positive selection during placental mammal evolution, suggesting involvement in a genetic conflict.
To identify the biological function of H2A.B, the authors produced a triple KO using CrispR/Cas9 technology (hereafter named H2A.B KO) and studied spermatogenesis of KO males. They showed that H2A.B KO alters the chromatin structure of postmeiotic male gametes (spermatids), leading to an increase in partially unwrapped nucleosomes, but does not visibly affect spermatogenesis. Finally, using a series of mating with parents of different genotypes, to discriminate between parental effect and zygotic effect of H2A.B, they studied KO effect on fertility and embryo viability and concluded that H2A.B is a paternal and maternal effect gene. The underlying mechanism is discussed but not addressed. Data indicate that the genetic conflict is not at the level of male gametogenesis (no sex ratio distortion was observed) but rather a parental conflict for post-fertilization resource allocation.**
We thank the reviewer for an accurate summary of our article: our manuscript is also the first to show that a eutherian mammal specific histone variant participates in a parental conflict for post-fertilization resource allocation (a conflict that first arose in the eutherian mammals).

****Major comments:**

(1) Data showing the validation and genotyping of the KO are lacking. This is essential for proper interpretation of all the data.**
We agree with the reviewer’s statement that this information is essential. We apologize for not including all the details necessary to properly validate our genotyping analysis; we have done so in the revision as we outline below.

**A) Only genotyping PCR bands are shown and it is not clear where the primers are located. Why isn't there a band amplified in the WT for H2afb3?**

We now include a schematic of primer locations in the context of all 3 H2A.Bs with their respective mutations (Supplementary Figure S1). In all 3 cases, the reverse primers are located 3’ of the ORFs. Due to the close homology of the H2A.B genes, we chose to use a forward genotyping primer specific for the H2afb3 knockout that spans the 15bp deletion (Supplementary Figure S1) specific to the targeted KO allele. This primer will not properly anneal in the case of the WT allele and so will not amplify a band. We have revised the methods section and figure legend to clarify this point.

**B) In the M&M section it is indicated that the authors '" used PCR genotyping to maintain our H2A.B triple KO line. For genotyping, a forward primer specific to the first deletion in the B1 allele was used (B1_mut_F: TTGCTGTGAGCCTGGTGGAGG; amplicon in mutant: 279bp, SupFig1B)". Is it what the 3rd panel shows in Fig S1B?**

This is correct. The third panel shows the mutant band amplified by this primer; no band is amplified in the case of the WT allele. We have edited the text to make this point more clearly in Supplementary Figure S1B.

**C) If so, please clarify and add details in the supporting legend. And it raises several questions...:**

We now include the requested details in the legend and revised Supplementary Figures S1 and S2. We thank the reviewer for pointing our omission.

**D) How did the authors test for deletion of H2afb3?**

As we indicate in the revised Methods, we used sanger sequencing of TOPO-TA cloned fragments amplified using the following primers:

H2afb3: B1_F:CAAGCCAAACTTTTCTTGAGGATGT, B1_R:TGAGCAGGTCAGCCAAGAAG;

H2afb2: B1/2_F:CAGGGAAAACTGTCTTCGAGA, B2_R:GATACTACTAAAGAGAATCAGCTTATAAAGAAGC;

H2afb1: B3_F:ACACGGAGAACTGTCTTCAAAG, B3_R:CAGAATCAAATACGGAAACATTTTTCCC.

These primer pairs allowed to identify and confirm the mutation of all 3 paralogs in the KO line (Supplementary Figure S2). These primers were sufficient for Sanger sequencing and identifying the molecular lesions. However, for genotyping ease, we used this information to design a KO-specific primer for the H2afb3 allele.

**E) If, once the triple KO lines were generated, only one PCR was performed, was it only maintained at the homozygous state to prevent recombination? During backcrosses ("we backcrossed heterozygous H2A.B/+ females to 'pure' C57BL/6J males for between 5 to 8 generations.") was the KO of the 3 alleles constantly monitored?**

We thank the reviewer for the opportunity to clarify the revision in response to this query. We note that given the close proximity of the three genes, a recombination event would be exceedingly rare. Nevertheless, we constantly monitored all three alleles during backcrossing and across our entire colony (experimental and maintenance). We indicate this fact in the revision and use one of these mating as a genotyping example in Supplementary Figure S1.

**F) Similarly in crosses using HET females, did the authors confirmed in the progeny, presence/absence of all 3 H3afb3 genes?**

Yes. As we now explicitly clarify in our revision, in crosses using HET females all 3 alleles are monitored.

**G) RTqPCR data would be useful additions to confirm that in triple KO animals H2afb is not expressed (from any of the 3 copies).**

We thank the reviewer for this suggestion. Since the CRISPR deletions are not located within the regulatory regions of *H2A.B* genes, we do not anticipate transcription to be affected in KO animals, just that they produce transcripts incapable of expressing intact protein. Complicating the design of RT-PCR analyses is the extensive gene conversion encompassing the ORFs and surrounding non-coding regions especially between *H2afb2* and *H2afb3* paralogs (as we previously described, Molaro et al. *Genome Research* 2018) and the fact that there are no annotated UTRs for these genes. Moreover, since all three genes are intronless, any frameshifts or premature stop codons would not necessarily trigger nonsense-mediated decay. This is why we focused on detailed DNA genotyping for our studies, where we could set primers outside the regions subject to gene conversion.

In response to the reviewer query, we have carried out RT-PCR analyses to try to distinguish WT and mutant paralogs on a gel. These experiments allowed us to see a truncated mRNA for *H2afb1* with no WT band, and a specific amplicon for the mutant allele of *H2afb3* (similar to our genotyping). However, no primer combination can distinguish *H2afb2* from *H2Afb3*. **If the editor requires**, we would be happy to include these panels in our revision.

**(2) Study of H2A.B KO effect on spermatogenesis. The authors conclude in this section that the KO does not impair spermatogenesis, but some additional experiments are needed to draw this conclusion. This is particularly important to understand the underlying mechanism of abnormal embryo development.**
We appreciate the suggestions made by the reviewer to strengthen our findings.

**(A) In the discussion the authors hypothesize that H2A.B could be involved in histone-to-protamine exchange. Yet, the authors do not satisfyingly address this question in the experiments they have performed. To determine whether or not histone-to-protamine transition is affected, one should investigate chromatin compaction of spermatozoa: simple tests such as aniline blue or CMA3 staining of spermatozoa would already give an indication. Western blot to compare histones and protamines levels in KO sperm with that of WT would also be a typical experiment to test this hypothesis.**

As suggested by the reviewer, we performed a new experiment where we carried out chromomycin A3 (CMA3) staining on 2 pairs of WT and KO male siblings (3 month old, not mated). CMA3 staining is a useful indication of protamine deficiency in sperm chromatin. We counted 200 sperm cells for each male and found that KO samples had fewer CMA3 positive cells than WT (rep1: 9% WT vs. 0.5%KO; rep 2: 3% WT vs. 1% KO). This result indicates that H2A.B KO sperm genome are less prone to deprotamination and might even be more compacted than WT. This also argues against a gross protamination defect in KO males. We have now included these findings in a new panel (Supplementary Figure S8E) and discuss these results in the revision.

Although we attempted to do western blots in these males to measure the levels of protamines deposited in sperm, we were unable to conclusively detect protamine 2 with a commercial antibody (Hup2 (P2) from Briarpatch @ 1:1000) from 1 million sperm cells in either WT or KO samples. We have therefore chosen to only include the CMA3 staining results in our revision.

**The authors present in their supplemental figure results from a comet assay to test for the presence of sperm DNA Damage. The experiment was repeated 3 times (each time 1 CTL vs 1KO) and the authors conclude that no damage are found despite the fact that in 2 out of 3 pairwise comparisons, the proportion of damaged cells was higher in KO than CTL. I think these results merit to be developed by adding more replicates.**
Following this comment from the reviewer, we added two more pairwise comparisons to the DNA damage assays (replicates rep4 and rep5). Replicate 4 showed little difference between WT and KO, whereas replicate 5 displayed high alkaline positive cells in in the KO compared to WT. We believe that this supports our conclusion that KO sperm show mild increases in apoptosis and/or DNA damage, but there is also great variation between replicates and no consistent differences were seen between the Comet and alkaline diffusion assays for DNA damage. We have updated Supplementary Figure S8D to include these additional replicates.

**(B) A recently published paper concluded that H2A.B KO (produced using TALEN strategy and on a different mouse background) results in mild spermatogenesis defects and male hypo fertility (Anuar et al 2019). For the sake of comparison it would be useful if Molaro et al could look at the same parameters as in the TALEN KO study, specifically the presence of "clogged tubules " (the authors did not observe "differences in cell types or seminiferous tubule organization between WT and KO" but it would be useful to show pictures to confirm that "clogged tubules" were not visible) and sperm morphology defects - such as malformed heads. Since the authors looked at sperm motility I am wondering if they saw flagella abnormalities as described in Anuar et al.**

We agree with this suggestion from the reviewer. We asked 2 pathologists to independently re-evaluate cross-sections of seminiferous tubules in WT and KO males (n=3). Neither of them found evidence of clogged tubules; both suggested that clogging can sometimes be an artefact of poor handling of samples during fixation leading to shedding of germ cells into the lumen and thus “clogging”. We now include pictures of WT and KO cross sections for comparison (Supplementary Figure S7A).

We also performed a systematic investigation of the sperm morphology in 4 sibling pairs of WT and KO animals used for motility and DNA damage analysis. Overall, there was no statistically significant differences in the proportion of sperm with abnormal heads, bent necks or abnormal tails between WT and KO (~86% with normal morphology, data now included in Supplementary Figure S7D) although there is higher variance in head shape in KO samples.

**In page 8 of their manuscript Molaro et al conclude that their findings on spermatogenesis are 'completely consistent with those reported for H2A.B KO in a recent study' (i.e. Anuar et al). I understand they mean that KO produced with a different strategy on different backgrounds can indeed give different phenotypes. I am not questioning this fact, but saying data in both studies are completely consistent is, to me, an overstatement. Especially, since number of pups/litter are significantly different in Anuar et al but not in Molaro et al.
So, to summarize, it would certainly improve the message of the manuscript if the authors could (i) perform additional experiments to rule out/detect any effect during spermatogenesis, (ii) rephrase their interpretation and hypotheses to be fully consistent with their data and data from Anuar et al.**
We agree with the reviewer that this was an overstatement. We were referring to the fact that in both cases no gross infertility effects were observed. We now conclude that while both studies found a mild effect on male fertility, our KO display fewer sperm and testis defects than in the Anuar et al. study. As noted by the reviewer, we hypothesize that strain background might explain most of these differences. We now include this discussion in revision.

**(3) The discussion could be shorten and the following points could be addressed/clarified:
- The part about H2A.B role in histone to protamine transition is speculative (see above, point 2A).**

In keeping with the reviewer’s comments, we note that the short discussion about histone-to-protamine transition is clearly presented as a hypothesis rather than result. Indeed, our CMA3 staining results allow us to clearly state that we do not observed effects of increased deprotamination in KO males; rather, they might have more compact chromatin packaging.

**- The authors claim that H2.AB is not present anymore in sperm cells based on mass spectrometry data by Baker et al 2008. This technique is probably not the most appropriate to identify persistent histone variants in spermatozoa. Before ruling out the hypothesis of H2A.B transmission from sperm to zygote, one could discuss that other approaches should be used to confirm that indeed H2A.B is not found in mouse sperm. NB. Since there is no good commercially available antibody I don't expect the authors to address this point in the present manuscript.**
We now add a citation to an additional study by El Kenanni et al., 2017, which didn’t detect H2A.B (although it did detect other short H2A variants) in elongating spermatids also using mass spec. approaches. However, we agree with the reviewer that we cannot rule out the possibility of small amounts of H2A.B retention in sperm; we now state that in our revision.

****Minor comments****

**(4) Presentation and interpretation of KO effect on embryo development

The way the fertility assay results are presented is a bit confusing. Some data are presented in the part "spermatogenesis": i.e. number of pups/litter in figure 2A, under the title "live animals at birth" and the rest is presented in the paragraph "H2A.B impacts embryo viability and fitness". Overall, these data together reflect fertility defects.**

We agree with the reviewer that these both relate to “fertility” although we were trying to make the distinction between fertilization and post-fertilization effects. Considering the additional experiments suggested by the reviewer, we have expanded the paragraph on spermatogenesis (including CM3A and Morphology) and dedicate a single paragraph ‘Loss of H2A.B affects male reproductive fitness” to fertility which includes all of Figure 2.

**The authors claim that "The most rigorous test of an effect of H2A.B loss on spermatogenesis is a fertility assay". But IVF or ICSI would be more appropriate to really test the ability of sperm to fertilize, and check effect on early embryo development, i.e. before implantation.
The question of embryo viability is addressed by measuring the % of resorbed embryo at one time point (E14.5); data indicate a trend toward higher % in WTxKO or KOxWT mating but only in KOxKO mating does it reach significance. Ideally, those measures should be done throughout gestation via ultrasound scan for instance, to determine at which stage(s) embryo development is affected by parental loss of H2A.B. Data would likely be significant and explain the observation that KO have "lower success in impregnation compared to WT males". It could maybe also help explain the observed perinatal lethality.**

**Neither IVF or ultrasound measures are trivial experiments to perform but, if possible, they would certainly strengthen the message of the paper, and clarify the discussion.**
We thank the reviewer for this important suggestion. Even though this was a minor comment, we invested significant time and effort to directly address the reviewer’s point in our revision. We performed in-depth IVF analysis to investigate the progression of pre-implantation embryos sired from KO and WT males. We *in vitro* fertilized WT oocytes with sperm from either KO or WT males (in triplicate with ~120 oocytes per replicate) and found no significant difference in average fertilization success (WT=66% vs. KO=71%) or progression through the blastocyst stage (new Figure 2C). **This is an important result and provides the strongest support for our contention that spermatogenesis is mostly normal in H2A.B KO males and that H2A.B KO sperm are capable of fertilization**. In combination with our subsequent findings, this result supports the hypothesis that H2A.B loss mostly affects post-implantation survival. We have modified the text and figure to account for this novel observation. We have also added a new co-author Dr. Priti Singh who carried out the IVF analyses.

As the reviewer points out, a detailed ultrasound is outside the scope of our revisions but could be the subject of a future study.

**(5) Cut&Tag analysis could be more developed.
(A) For peak calling the authors used SEACR which produces fewer peaks than other peak callers (because more stringent). Did the authors compared with other peak callers or less stringent parameters to see if they obtain similar results?**

Previous reports including by the pioneers of the technique have remarked about how peak-callers like MACS2 cannot properly process CUT&RUN signals due to sparse data (Meers et al., *Epigenetics Chromatin* 2019). Indeed, we found 3x fold more peaks using MACS2, most of which were false positives. Thus, we preferred to use the more conservative but reliable SEACR analyses.

**(B) Since H2A.B has been shown to be enriched on the X chromosome, did the authors look in their CUT&TAG experiments whether differences were observed for the sex chromosomes compared to autosomes?**

We looked at the fraction of reads mapping to the sex chromosomes vs. autosomes and found no difference in their relative ratios. We now report this result in Supplementary Figure S4E.

**(C) Presenting IGV or UCSC screen shots showing representative regions could be useful.**

We agree. We picked a region with representative peaks and report this as a panel in Supplementary Figure S11.

**(6) Other minor comments:
- Typo Page 4 in the following sentence: "Consequently, imprinting defects are result in reduced embryo fitness ...."**

We have corrected this typo and thank the reviewer for pointing it out.

**- In the "germ cell spreads" part of the M&M, info are presented for male and female germ cells but only data from male germ cells appear in the manuscript.**
We thank the reviewer for pointing this out. Following reviewer 2 suggestion (Major Comments, point 3) as well, we now include preliminary results for H2A.X staining performed on E17.5 females germ cell spreads (Supplementary Figure S10). These show no discernible differences between WT and KO pachytene cells.

**(7) Suggestions to improve data presentation (in figures):
- Figure 1G: "partially unwrapped nucleosomes" could be written under the right panel to make it self-explanatory**

We have modified this figure accordingly. **- Fig 1G legend please explain what arrowheads stand for (sites cut by Mnase)**

We have added this to the legend. **- Underneath figure 3A - maybe add male and female symbols**

We have modified this figure as suggested. **- I am a bit disturbed by X* symbol (because it can mean something different, cf. Mus minutoides) - to avoid confusion it would probably be better if it was changed to XKO or Del**

We replaced X* by X^KO^ as suggested by the reviewer. **- It would be clearer to the readers if, in FigS2 and 4 legend, instead or in addition to Punnett square was written sex ratio of the progeny**

We have made this change in new Supplementary Figure S9.

**For next time, please add line numbers to facilitate the review process.**

We have added line numbers in our revision. We apologize for this omission in our original submission.

**Reviewer #1 (Significance (Required)):**

**The results are highly significant and novel since they indicate a paternal and maternal effect of H2A.B on embryo development, which was not foreseen. Another KO of H2A.B genes (using a different strategy and different mouse background) was recently published and concluded on male subfertility and global effect of H2A.B KO on pre-mRNA splicing, in spermatids (Anuar et al 2019). The manuscript by Molaro et al provides a different and interesting perspective on the biological role of H2A.B. Both studies pave the way for further analyses of H2A.B molecular role and impact on spermatozoa chromatin organization and RNA content. It will also be interesting to investigate whether other short H2A variants could have similar effects.
The manuscript is for a broad audience including scientists of the chromatin biology, reproductive biology as well evolutionary genetic fields. It could probably be re-organized as a report, if needed.**

**My expertise are: spermatogenesis, chromatin biology, sex chromosomes and intragenomic conflicts.**
We would like to thank the reviewer for the appraisal of our work and for highlighting both its novelty and high significance. Their comments and suggestions have greatly improved our manuscript. We also agree with the reviewer that both H2A.B KO studies are complementary and together help provide more information on this class of histone variants.

**Reviewer #2 (Evidence, reproducibility and clarity (Required)):

**Summary:**

H2A.B genes encode fast evolving testis-expressed histone variants whose function in placental mammals is still poorly understood. Previous work (Anuar et al, 2019), using TALENS-based knock-out, has suggested that H2A.B genes are required for male fertility and normal spermatogenesis, with an increase in the proportion of abnormal sperm and clogged seminiferous tubules. They showed that the dynamics of transition protein 1 incorporation is abnormal after H2A.B knock-out. Since transition proteins are important for histone replacement by protamines, the authors of the previous manuscript propose this is a likely explanation for the observed defects in male fertility. Interestingly, it was also observed increased levels of H3K4me3 in round spermatids and significant changes in gene expression.

In the current manuscript, and using CRISPR-Cas9-mediated genome-editing, Molaro et al further expand the observed phenotypes after H2A.B knock out. Significantly, they showed that H2A.B encodes novel parental-effect genes whose function is required in the mother and in the father, but not in the embryo, for embryonic viability and growth during gestation. Interestingly, and consistent with their previous result suggesting a function in the mother, Molaro et al detected H2A.B expression in the female germ cells at the onset of meiosis I.

Finally, and although they could detect altered chromatin structure, with genome-wide nucleosome unwrapping, after H2A.B knock-out, they failed to detect significant defects during spermatogenesis, without changes in the rate of production and motility of sperm, or defects in the seminiferous tubule organization.**
We thank the reviewer for this summary of our findings and highlighting both the paternal-effect and female germ cell expression as novel findings.

**Major comments:**


**1) The CRISPR-Cas9 strategy used by these authors was based in the deletion of parts of the three genes that encode the fold domain of H2A.B histone. Despite the Sanger sequencing of H2A.B deletion alleles, mRNA and protein levels of H2A.B were not determined after H2A.B deletion. This is important to ensure they are bona fide null alleles, and eventually control for the expression of truncated/ abnormal proteins generated by the H2A.B frameshift mutations.**
In response to Reviewer 1 comments, we have expanded and clarified our DNA genotyping results. We have also found evidence of production of truncated transcripts for two of the three mutant genes, whereas we cannot distinguish the third (see reviewer 1, point 1G). Without any working commercially available antibodies, we cannot verify our KO at the protein level. We cannot rule out the possibility of production of truncated proteins being produced in the KO males, but these would be entirely missing their histone-fold domains; in that respect, we are confident these are *bona fide* null alleles for chromatin function. We note that the Anuar et al., 2019 study which reported TALEN-mediated deletions in H2A.B also relied on internal deletions in the coding regions.

**2) The authors should discuss in detail the fact that they failed to detect significant spermatogenesis defects; contrary to what was recently reported by Anuar et al., 2019.**

We agree with the reviewer’s suggestion. We now include a detailed discussion of these differences in the main text. First, we point that these differences are likely due to strain background (*FVB/NJArc* vs. *C57BL/6J*) and incomplete penetrance of the phenotypes. To allow a full comparison with the Anuar study, we have added an analysis of sperm morphology and additional analysis of testis histology (Supplementary Figure S7A). While we there are differences regarding these phenotypes, we now highlight that we similarly report mild fertility defects for KO males; however, in our strain background these fertility defects (in terms of litter sizes) are not significant.

**3) Since the authors report H2A.B expression in the female germ-line they should explore potential phenotypes in the germ-line of KO females - for example, is female meiosis and oocyte development normal in these mutants?**

Following this suggestion, we performed germ cell spreads on 2 KO and 2 WT females at E17.5 and stained for gammaH2A.X and SYCP3 (Supplementary Figure S10). We found no significant defect in meiotic progression and found no defects in H2A.X staining at this stage. We suggest that, as seen for males, H2A.B loss doesn’t seem to affect female meiotic progression. However, more experiments will be needed in the future to fully understand the novel function of H2A.B in females. We note that our study is the first to find that H2A.B is expressed as a maternal effect gene in female germ cells. Understanding the mechanistic basis of this contribution is going to be the topic of a future study at least on the same scale as this study.

**4) In SupFig2C, there is an approximate 10% increase of litters with at least one dead pup in WT x KO genotype, but not in KO x KO genotype. Authors should clearly provide an explanation for these results, as they are inconsistent with the proposal that H2A.B are required in the father and in the mother for normal viability and growth of the embryo.**

The reviewer raises an interesting point of discussion. Chief among those is the fact that any embryos that are resorbed during gestation will not be counted in the litter at birth. Thus, the timing of manifestation of the paternal versus paternal-maternal effects of H2A.B may explain the difference in liters with one dead pup. We incorporate this suggestion into our discussion about the asymmetry in paternal and maternal contributions of H2A.B.

**5) Authors argue that the mild decrease in the % labyrinth area of the placenta may explain the reduced weights of KO embryos (SupFig6). However, the sample size of these results (n=2; two males for wild-type and one female and one male for KO) is clearly too small to make such conclusion. If authors want to suggest such possibility, which is definitely interesting, they should significantly increase sample size. They should also comment the mild increase in the % of junctional area in KO embryos.**
We agree with the reviewer that our data size is too small to make a meaningful conclusion. We have, therefore, removed the discussion of placental phenotypes from this revision. Future experiments will be needed to address this specific effect.

****Minor comments:**

1) In the PCR genotyping of the H2A.B deletion alleles (SupFig 1B), it is unclear why the wild-type control band is absence from the H2afb3 variant.**
We apologize for the lack of clarity. Reviewer 1 also had this query. Our forward primer is designed to span the H2Abf3 deletion and therefore does not anneal to the WT genome. We clarified this point in the text and included a detailed figure of the genotyping primer in the context of each deletion in the revised Supplementary Figure S1.

**2) In SupFig. 5C, there is an apparent increase in sperm motility in the KO condition. Although this result is most likely not statistically significant due to the standard variation, it is nevertheless surprising. Authors should clearly indicate sample size, check for statistically significance, and clearly comment for the increased standard deviation of sperm motility in KO mice.**

We thank the reviewer for this suggestion. The sperm motility was evaluated on 100 caudae-isolated sperm cells across each 3 WT and 3KO males used in our fertility assay. As noted, the standard deviation from the mean is quite higher in the KO. This is due to one KO sample with higher (>70%) motility than the rest of the samples (~50%). However, the two-tailed t-test comparing is not statistically significant. We now include these details in our revision.

**3) In SupFig5G, DNA damage results are not consistent among replicas. Why is this the case? Authors should comment on this.**

Following reviewer 1 suggestions, we included 2 more replicates which also displayed significant variation (Supplementary Figure S8D). We attribute these inconsistencies to the incomplete penetrance of the DNA damage phenotype, which is also reflected in differences between the Comet and alkaline diffusion assays.

**4) Recurrent typing error: instead of "testes" should be "testis"**
We appreciate the reviewer suggestion and kept testes when plural and testis when singular.

**Reviewer #2 (Significance (Required)):

Altogether this manuscript gives a relevant contribution for a better understanding of the developmental functions of this class of histone variants. The observation that H2A.B encodes a parental-effect gene whose function is required in the mother and in the father for normal viability and growth of the embryo is definitely interesting, and potentially highly relevant.

Yet, a mechanistic explanation of the observed embryonic phenotypes is still lacking. In addition, and given its expression, the potential (meiotic) functions of H2A.B in the female germ line are not explored. Finally, it is also unclear why the spermatogenesis defects recently reported by Anuar et al. 2019, were not reproduced in this manuscript.**
We would like to thank again reviewer 2 for their positive comments. We are grateful for their appraisal of our work as interesting and relevant. We have addressed in more details the discrepancies between the Anuar et al., study and ours including more experiments regarding spermatogenesis and sperm function. Understanding the mechanistic basis of the embryonic phenotypes and the female germ cell biology is beyond the scope of our current study, which (as the reviewer notes) has **several novel, unprecedented findings for a histone variant.**

**Reviewer #3 (Evidence, reproducibility and clarity (Required)):

This study addresses the role of short histone H2A variant, H2A.B in mice. H2A.B is interesting to study, because it is rapidly evolving in contrast to the well-conserved canonical histone genes and also has unusual biochemical property (e.g., faster exchange rate on chromosomes). The authors combined the high resolution chromatin profiling technique, CUT&RUN and CRISPR genome-editing to demonstrate that H2A.B knockout males have altered chromatin structure across the genome in post-meiotic cells. But the more interesting result is that embryos from H2A.B knockout parents showed increased mortality rate. Further, the authors discovered that the genotype of both father and mother, but not zygote, is critical to support embryonic viability, identifying novel parent-effect gene in mice.**
We are grateful for the positive appraisal and many helpful comments of the Reviewer, which have helped our revised narrative.

****Major concerns:**

The data presented is convincing, strongly supporting the authors' conclusions, and I only have a couple of comments that could be addressed by writing.**
Once again, we are grateful for the positive comments and the constructive suggestions to improve our paper.

**1) As one of the major findings in this study is the increased inviable conceptuses from crosses of H2A.B KO parents (Figure 3A), I suggest the authors to define the "severely delayed" embryos and show representative images of resorbed and severely delayed embryos in the figure.**

This is an excellent suggestion. Unfortunately, our IACUC approval for animal procedures did not permit us to take pictures of resorbed embryos at the time of dissection. During the several months of data collection, we were also unsuccessful at extracting DNA for sexing and genotyping from resorbed embryos.

We now more clearly define what we mean by ‘severely delayed embryos’; these correspond to embryos which were implanted and visible but displayed no visible vascularization and delayed organ development compared to stereotypical E14.5 conceptuses. These were collected and genotyped. We now discuss this in the method section of the revised manuscript.

To complement our analysis of embryo viability, in response to Reviewer 1’s suggestion, we were able to include a new comparative analysis of *in vitro* fertilized eggs up to blastocyst stage from WT and KO males (Figure 2C). Eggs fertilized by WT or KO sperm developed normally in similar proportions supporting the hypothesis that no discernible defects are observed at fertilization or pre-implantation; all defects appear to arise following implantation.

-**On page 15, the sentence "Although maternal H2A.B does not act as a 'demand-inhibitor' per se, it ensures equitable resource allocation to all embryos by increasing fetal weights, independent of whether they are sired by WT or DH2A.B males" is counter-intuitive. Shouldn't reasonably decreasing fetal weight of a litter allow the mother to produce more litters overtime? And how does increasing fetal weights allow for equitable resource allocation? Is the increased weight supposed to diminish weight differences produced by different paternal genotypes? Please explain and provide references if possible.**

We agree with this concern from the reviewer about the previous version, which may have led to confusion by combining two ideas. We now make clear that it is the last possibility that we favor: maternal H2A.B acts as a capacitor by diminishing weight differences produced by different paternal genotypes. We have extensively revised this to make clear that H2A.B only partially meets the canonical parental antagonism model because maternal H2A.B is not a ‘demand-inhibitor’. We also point out that paternal and maternal contributions are not symmetric thus paternal and maternal optima for H2A.B are different, in keeping with the parental antagonism principles. We are grateful for the opportunity to clarify this and would be very eager to hear from the reviewer whether this addresses their concern.

**-On page 15, referring to the sentence "Because of their X-linkage, any male-beneficial (demand enhancer) allele of H2A.B would be fully exposed to selection in hemizygous males, but its female-deleterious effects could be masked in the heterozygous females (Rice 1984)."; Could you discuss how this scenario is altered when considering a gene that exhibits both maternal and paternal effect as well as a haplo-insufficient allele in females (which seems like the female-detrimental effects are not masked). Is there an evolutionary explanation as to why the same gene would evolve both maternal and paternal effect? And are there any references (in addition to Rice 1984) you could use to support this part of the discussion?**

We add to this discussion by pointing out that male-beneficial alleles will likely sweep through faster than female-beneficial alleles due to haploinsufficiency of the latter. We also add that it is rather unexpected, indeed perplexing that maternal H2A.B might act as a suppressor itself. We know of no precedent where the same gene acts as both a paternal and maternal effect locus. We are a bit hesitant to expand this section of the Discussion, which was already criticized by one reviewer for being lengthy but we have added some additional references in support of the idea.

****Minor concerns:**

1) On page 4, in the sentence "Consequently, imprinting defects are result in reduced embryo fitness and poor implantation outcomes but ..." remove the extra are.
2) On page 7, in the 1st sentence of the second paragraph, "H2AB KO" is missing the period between A and B.**
We have corrected these typos. Thank you.

**3) On page 7, referring to the last paragraph before the "dH2A.B does not impair spermatogenesis" section; I am perplexed by this and it could use a little more explanation. If "H2A.B loss leads to widespread nucleosome unwrapping" then wouldn't you expect H2A.B KO to have more mono-nucleosomes with smaller footprints as a result? How does widespread nucleosome unwrapping lead to an absence of smaller footprints?**

We thank the reviewer for giving us the opportunity to clarify this finding and explain it better in the revision. This is especially important since there is no accepted language to describe these observations. We now include a new Supplementary Figure S5 schematic to outline the various nucleosomal fragments we expect to see in WT and KO animals:

1) The nucleosome unwrapping described in Figure 1 leads to the formation of small fragments that are much smaller (≤80bp) than mono-nucleosomes (≥150bp). We hypothesize these to be the result of transition structures which might contain transition proteins and/or protamine.
2) The smaller mono-nucleosome footprints refer to the fact that H2A.B containing nucleosomes have been reported to have less wrapping (~120bp) than core nucleosomes (~150bp) (e.g. Bao et al. 2004). As expected from these prior studies, we find that in H2A.B KO spermatids, mono-nucleosomes fragments have footprints that are closer to core size, i.e., slightly larger than in the WT case, because the ‘smaller’ H2A.B containing nucleosomes are lost.

We have clarified some of this explanation in the discussion: “Closer examination revealed that the sub-nucleosomal fragments do not correspond to specific genomic locations but rather represent a genome-wide pattern of partially unwrapped nucleosomes. This finding might be surprising since H2A.B-containing nucleosomes have been shown to have smaller MNase footprints also suggestive of unwrapping (Arimura et al. 2013; Bao et al. 2004; Syed et al. 2009). However, the sub-nucleosomal fragments we have recovered are much smaller (≤80bp) than the footprints reported for H2A.B nucleosomes (~120bp). Furthermore, corresponding to the loss of H2A.B-nucleosomes, we found that mono-nucleosomes fragments released by CUT&RUN are indeed larger in KO than WT chromatin”

**4) On page 13, referring to the sentence "Even though H2A.B is itself not deposited in mouse sperm (Baker et al. 2008), a previous study found H2A.B staining in the first cleavage cycles of mouse zygotes (Wu et al. 2014)."; could the H2A.B staining in the first cleavage cycles be a result of maternally deposited H2A.B?**

We agree with the reviewer. It is possible that this staining corresponds to maternally deposited H2A.B incorporated into chromatin. We gratefully include this possibility in this revised version.

**Reviewer #3 (Significance (Required)):

This study provides a conceptual advance in the genetic, epigenetic, and evolutionary biology field. The authors have identified the first biparental-effect histone variant gene in mammals. It is especially intriguing that histone H2A.B has paternal effect on embryo development, since sperms get rid of most histones including H2A.B. Further, the authors revealed the hitherto unknown biological functions of H2A.B in embryo growth and viability. As an evolutionary cell biologist, I am convinced that this study has significant impacts on the genetic, epigenetic, and evolutionary biology field.**

We would like to thank reviewer for their support and appraisal of our work. Their insights greatly improved our manuscript. We are also grateful to the reviewer’s comment finding our work novel, interesting and impactful to a broad audience.
