# Supplementary material for: Biparental contributions of the H2A.B histone variant control embryonic development in mice
Source: PLoS Biol. 2020 Dec 23;18(12):e3001001. doi: 10.1371/journal.pbio.3001001 (PMC7757805; doi:10.1371/journal.pbio.3001001)
Supplement: Attachment — Submitted filename: ResponseToReviewers.docx [file pbio.3001001.s027.docx]

**Editor**

**We're delighted to let you know that we're now editorially satisfied with your manuscript. The reviews are attached below and you will see that they are all very positive without further requests. Nevertheless, Reviewer 2 would like you to add some discussion of a potential mechanistic explanation for the embryonic roles of H2A.B, but we will leave it up to you to decide.**
Thank you for your handling of our manuscript. As we respond to Reviewer 2’s comment, the post-implantation phenotype is novel enough that we feel that speculating on the mechanism would be premature at this stage, but we agree that this is an excellent line of inquiry to now pursue.

**I would also like to encourage you to submit one of the pachytene cells images shown in Fig. S10 as potential cover image.**

We have now uploaded two images as a ‘Striking Figure’. However, we would suggest not using either of these images as we found no differences of H2A.B loss on either of these phenotypes.

**Rev. 1:
In the revised version of their manuscript, the authors have satisfyingly addresses the issues/questions that I had raised in my first review. The study is novel and highly significant; I support its publication without additional changes.
NB. I detected two typos: one in Fig S4E title, the other Line 492 (missing word in sentence "Our second novel finding is that H2A.B expressed during female meiosis").
And while re-reading my review I realize I wrote "CUT&TAG" instead of Cut&Run, my apologies for the confusion!**
We thank the reviewer for their kind comments. We have corrected the typos and are grateful to the reviewer for pointing these out.

**Rev. 2:
This is a significantly improved version of the manuscript previously submitted to Review Commons. The authors have successfully addressed most of my comments.
This is a highly relevant work, giving an important contribution for a better understanding of the developmental roles of short H2A histone variants in placental mammals.
Given its relevance to the field, I recommend this manuscript in its current format for publication in PLoS Biology. Yet, the lack of a precise mechanistic explanation for the embryonic roles of H2A.B, should nevertheless be considered before a final editorial decision.**
We thank the reviewer for their comments. Although H2A.B’s role in genome-wide nucleosome wrapping during spermatogenesis is revealed by our analysis, we agree with the reviewer’s comment that a mechanistic explanation for H2A.B in embryonic development will require additional tools and analyses. We feel that speculating on a mechanism that would manifest post-implantation would be premature.

**Rev. 3:
I went through the revised manuscript and feel that the manuscript is now acceptable.**

We thank the reviewer for their earlier comments that allowed us to significantly revise our Discussion.
